# GP88 (PC-Cell Derived Growth Factor, progranulin) stimulates proliferation and confers letrozole resistance to aromatase overexpressing breast cancer cells

**DOI:** 10.1186/1471-2407-11-231

**Published:** 2011-06-09

**Authors:** Tesfom Abrhale, Angela Brodie, Gauri Sabnis, Luciana Macedo, Changsheng Tian, Binbin Yue, Ginette Serrero

**Affiliations:** 1 A&G Pharmaceutical Inc. 9130 Red Branch Rd. Columbia, MD, USA; 2 Department of Pharmaceutical Sciences, University of Maryland School of Pharmacy, 20 N Pine Street, Baltimore MD 21201, USA; 3 Department of Pharmacology and Experimental Therapeutics, University of Maryland School of Medicine, HSF-I, 685 W. Baltimore Street, Baltimore, MD 21201, USA; 4 Program in Oncology, Marlene and Stewart Greenebaum Cancer Center, University of Maryland School of Medicine, 22 S. Greene St. Baltimore, MD 21201, USA

## Abstract

**Background:**

Aromatase inhibitors (AI) that inhibit breast cancer cell growth by blocking estrogen synthesis have become the treatment of choice for post-menopausal women with estrogen receptor positive (ER^+^) breast cancer. However, some patients display de novo or acquired resistance to AI. Interactions between estrogen and growth factor signaling pathways have been identified in estrogen-responsive cells as one possible reason for acquisition of resistance. Our laboratory has characterized an autocrine growth factor overexpressed in invasive ductal carcinoma named PC-Cell Derived Growth Factor (GP88), also known as progranulin. In the present study, we investigated the role GP88 on the acquisition of resistance to letrozole in ER^+ ^breast cancer cells

**Methods:**

We used two aromatase overexpressing human breast cancer cell lines MCF-7-CA cells and AC1 cells and their letrozole resistant counterparts as study models. Effect of stimulating or inhibiting GP88 expression on proliferation, anchorage-independent growth, survival and letrozole responsiveness was examined.

**Results:**

GP88 induced cell proliferation and conferred letrozole resistance in a time- and dose-dependent fashion. Conversely, naturally letrozole resistant breast cancer cells displayed a 10-fold increase in GP88 expression when compared to letrozole sensitive cells. GP88 overexpression, or exogenous addition blocked the inhibitory effect of letrozole on proliferation, and stimulated survival and soft agar colony formation. In letrozole resistant cells, silencing GP88 by siRNA inhibited cell proliferation and restored their sensitivity to letrozole.

**Conclusion:**

Our findings provide information on the role of an alternate growth and survival factor on the acquisition of aromatase inhibitor resistance in ER^+ ^breast cancer.

## Background

Estrogen plays a crucial role in breast and endometrial carcinomas in addition to being involved in normal physiological processes [1]. The primary site of estrogen biosynthesis in premenopausal women is the ovaries [2]. After menopause however, peripheral adipose tissue becomes the main source of estrogen synthesis [2,3]. The principal enzyme responsible for the conversion of androgens to estrogens is the cytochrome P450 complex known as aromatase (CYP 19) [1,2]. Several studies have demonstrated that this *in situ *estrogen production plays a more important role than circulating estrogen in breast tumor growth [4,5]. Estrogen produces a variety of cellular responses, such as stimulation of cell proliferation, inhibition of apoptosis, and enhancement of various peptide growth factor/growth factor receptor expression in ER^+ ^breast cancer cells [2,3].

Treatment options for patients with hormone-dependent ER^+ ^breast cancers are estrogen antagonist such as tamoxifen, estrogen receptor down regulator such as Fulvestrant or inhibitors of estrogen biosynthesis such as aromatase inhibitors [6,7]. Tamoxifen has been the major agent used to inhibit breast cancer growth until the development and clinical application of estrogen receptor (ER) antagonists such as ICI 182,780, also called Faslodex or Fulvestrant [6]. Aromatase inhibitor is now the treatment of choice for post-menopausal breast cancer patients. The inhibitory effect of anti-estrogen therapy is observed almost exclusively in breast tumors that are ER^+^. However, after prolonged anti-estrogen therapy, breast carcinoma often progress and become estrogen-insensitive and refractory to treatment [8,9]. It is widely documented that the inappropriate activation of growth factor signaling cascades, either through overexpression of growth factor, or via up-regulation and increased activation of their target growth factor receptors or their recruited downstream signaling elements, can readily promote anti-hormone failure in breast cancer cells [10-12]. This phenomenon was demonstrated for the overexpression of multiple growth factors and their receptors, including heregulins acting through HER3 and HER4 [13,14], epidermal growth factor [15] and transforming growth factor (TGF)-α acting through the epidermal growth factor receptor (EGFR) [16,17], insulin-like growth factors IGF-I and IGF-II acting through the IGF-IR [18,19], and HER2 receptor contributing to anti-hormone failure either directly when overexpressed [20-23] or indirectly through heterodimerization with other erbB receptor family members [17].

Increased autocrine or paracrine growth factor signaling network could then bypass the need for ER-mediated growth stimulation in human breast cancer cells and would make anti-estrogen therapy ineffective. Such an elevated or sustained growth factor signaling within the anti-estrogen-resistant models would eventually lead to endocrine insensitivity, or to ER signaling being circumvented [10-12]. Autocrine and paracrine growth factor signaling cross-talk with estrogen receptor (ER) signaling to facilitate tumor growth [24]. Therefore, increase in autocrine growth factor signaling that mediates proliferation signals may induce resistance to endocrine therapy [25].

One autocrine growth factor under study in our laboratory and that has been implicated in the development of tamoxifen resistance and estrogen independence is PC-cell Derived Growth Factor (GP88) [26]. GP88 also called granulin/epithelin precursor or progranulin, is the largest member of a unique family of growth modulators characterized by 7.5 cysteine-rich 6KDa polypeptide repeats [27-29]. Published reports have shown that GP88 was expressed in human breast cancer cells in a positive correlation with tumorigenesis, and estradiol (E2) stimulated GP88 expression transcriptionally in ER^+ ^cells [26]. Inhibition of GP88 expression by antisense GP88 cDNA transfection in human breast adenocarcinoma MDA-MB-468 cells led to inhibition of tumor formation in vitro and in vivo in mouse xenograft studies [30]. In pathological studies of 203 human breast cancer biopsies, GP88 was expressed in 80% of invasive ductal carcinomas in correlation with parameters of poor prognosis [31] whereas it was negative in benign lesion and normal mammary epithelial tissues. In addition, circulating GP88 was found in serum of breast cancer patients at an increased level when compared to healthy volunteers [32]. GP88 overexpression in the human breast cancer cell line, MCF-7, conferred estrogen-independent growth, tamoxifen resistance and increased invasive properties [26,33,34]. Previous studies also demonstrated that tamoxifen-resistant MCF-7 cells, selected by culturing cells in the continuous presence of tamoxifen, expressed higher level of GP88 than tamoxifen-sensitive cells. This suggests that GP88 plays a critical role in breast cancer tumorigenesis and that there is a direct relationship between GP88 overexpression and tamoxifen resistance exists.

Based on the above information, it would be interesting to examine the effect of GP88 on breast cancer cells response to aromatase inhibitors. Using cellular model systems established from two independently derived MCF-7 cell lines overexpressing aromatase activity, we examined the effect of GP88 on letrozole responsiveness. One model consisted of the MCF-7-CA cell line obtained by transfection of an vector expressing aromatase cDNA [4] and its letrozole resistant counterpart (LTLT cell line) derived from an MCF-7CA tumor developed in mice treated with letrozole [23,35]. The second model included the aromatase overexpressing breast cancer AC1 cells [36] and letrozole resistant derivative AC1-LetR obtained by continuous cultivation of AC1 cells in the continuous presence of letrozole.

## Methods

The following reagents were obtained: Lipofectamine and Plus reagent from Life Technologies, Inc. (St. Paul, MN); agar and zeocin from InVitrogen, (Carlsbad, CA), androstenedione and β-Estradiol (E_2_) from Sigma (St. Louis, MO);, DME/F12 nutrient medium and G418 from Gibco, (La Jolla, CA), fetal bovine serum (FBS) from Hyclone, (Salt Lake City, Utah); CellTiter-Glo Reagent from Promega, (Pittsburgh, PA); GP88 SiRNA and control SiRNAs (smart pool duplex), from Dharmacon (Chicago, Il). Letrozole was provided to Dr. Angela Brodie by Novartis. Purified GP88 and all anti-GP88 antibodies and EIA kit to measure GP88 expression were developed by the Precision Antibody Division of A&G Pharmaceutical.

### Cell lines and culture condition

Human ER^+ ^breast cancer cells lines MCF-7CA, MCF-7AC1, LTLT-CA and UMB1-CA cell lines were derived from MCF-7 cells stably transfected with aromatase cDNA [35,36]. MCF-7AC1 were maintained in DMEM/F12 medium supplemented with 5% FBS in the presence of 650 μg/ml G418. LTLT and UMB1 cells [35] were cultured in steroid-depleted medium consisting of phenol red-free (PRF) DMEM/F12 medium supplemented with 5% charcoal-stripped (ChX) FBS (PRF-ChX medium) in the presence of 750 μg/ml G418. In addition, LTLT were cultivated in the presence of 1 μM Letrozole.

### Anchorage-independent growth in Soft Agar

Cells were plated in 6-well plates at 1 × 10^4 ^cells per well in 0.33% agarose in PRF-ChX medium layered on top of 0.6% agarose in the same medium with the indicated treatments. Colony formation was observed after 14-21 days in culture. Following staining with 0.005% crystal violet, colonies were counted under a microscope.

### Cell proliferation assay

Cells were plated at 2 × 10^3 ^cells/well in 96 wells plate in PRF medium supplemented with 5% ChX FBS (PRF-ChX medium). The next day, medium was removed and PRF medium supplemented with the intended treatments and growth factors was added and incubated for specified times. Cell growth was measured using CellTiter-Glo Reagent following manufacturer's instructions (Promega). Recombinant human GP88 produced in CHO cells was prepared and affinity purified in our laboratory as described previously [26,33,34].

### GP88 cDNA Transfection in MCF-7AC1 cells

Stable transfection of MCF-7AC1 cells with plasmid DNA of pSectag expression vector containing human GP88 cDNA [26] was conducted using lipofectamine and Plus reagent according to manufacturer's protocol. After transfection, selection of transfected clones was performed in the presence of 80 μg/ml of selection drug Zeocin. After two weeks, single clones were picked and tested for GP88 expression by immunoprecipitation/western blot as described previously [26] and GP88 production in culture medium with GP88 EIA kit developed in our laboratory.

### Aromatase activity assay

Cells were plated at a density of 1.5 × 10^5 ^cells/well in 6 wells plate in PRF-ChX medium. The next day, cells were washed with PBS and PRF medium supplemented with 1% ChX FBS (no G418). Radiolabeled [1 beta^3^H]-androstenedione (23.5 Ci/mmol, Perkin-Elmer) was added and incubated for the indicated times. Tritium released from the C-1beta during aromatization to form ^3^H_2_O was then measured in the supernatant [34], after addition of trichloroacetic acid to stop the reaction, centrifugation, and addition of chloroform. After centrifugation, 700 μL of the upper phase was transferred to a tube to which 700 μL of 2.5% activated charcoal was added and spun once more. Then 700 μL of the supernatant was taken and 4 ml scintillation liquid was added and the radioactivity was measured in a liquid scintillation counter.

### Silencing of GP88 by siRNA in Letrozole resistant cells

LTLT cells or AC1-LetR cells (5,000 cells/well) were plated in 96 wells plate overnight in ChX-PRF medium. The next day transfection of GP88 or control SiRNA (smart pool duplex) (1 μM) was carried out following the protocol of the manufacturer (DHARMACON). Cell viability was measured by cell Titer-Glo assay and the level of GP88 was measured both by western blot analysis and EIA as indicated below.

### Measurement of GP88 expression by Western Blot analysis and by EIA

GP88 expression in breast cancer cells and culture media was measured using two methods: Western blot analysis and EIA. For western blot analysis of GP88 expression in cell lysates, cells treated with GP88 or control siRNA (1 μM) were washed with ice cold PBS twice and lysed with RIPA buffer containing protease inhibitors (150 mM NaCl, 50 mM Tris-HCL, 1% NP-40, 0.25% Na-Deoxycholate, 1 mM EDTA, 1 μg/ml Aprotinin, Leupeptin, and Pepstatin, 1 mM Na3VO4, 1 mM PMSF and 1 mM NaF. 50 μg of total protein lysate were analyzed by 10% SDS gel electrophoresis. Western blot analysis was performed as described previously [26]. For Western blot analysis of GP88 in culture medium, conditioned medium normalized to cell number was collected and brought up to 1 ml volume and incubated with 1 μg of anti-human GP88 rabbit polyclonal antibody in the presence of 30 μl of protein G agarose overnight at 4°C. The next day, the agarose beads were spun and washed three times with ice cold PBS and resuspended in 40 μL of 2x sample buffer to perform SDS-PAGE on a 7.5% polyacrylamide gel followed by western blot analysis using anti-human GP88 mouse monoclonal antibody also developed in our laboratory [26]. Quantification of GP88 in culture medium was also carried out using an GP88 EIA kit developed in our laboratory.

### Determination of Bcl-2 expression in response to GP88 and letrozole

The ability of letrozole to down regulate bcl-2 expression was examined for MCF-7-AC1 cells. AC1 cells were plated at 1.5 × 10^5 ^cells in 35 mm plate and incubated in PRF medium with 5% ChX-FBS (ChX-PRF). The next day, the medium was removed and fresh medium was added with the treatments: 25 nM of androstenedione (AD) or AD with 5 nM letrozole (Let) in the absence or presence of GP88 at the indicated concentrations. Control corresponded to cells cultivated in ChX-PRF. 48 hours later, total RNA was extracted using Trizol™ reagent (InVitrogen) and resuspended in DEPC-treated water for measuring bcl-2 and GAPDH mRNA expression by RT-PCR. PCR conditions and the oligonucleotide sequences of the specific forward and reverse primers for bcl-2 and GAPDH have been described previously [33].

### Statistical Analysis

All experiments were performed in triplicates and repeated at least three times. Data are expressed as mean with standard deviation. Student's t-test was used to determine the significance of results with p < 0.05 as statistically significant.

## Results

### GP88 induces cell proliferation and confers letrozole resistance *in vitro*

**Figure 1:**
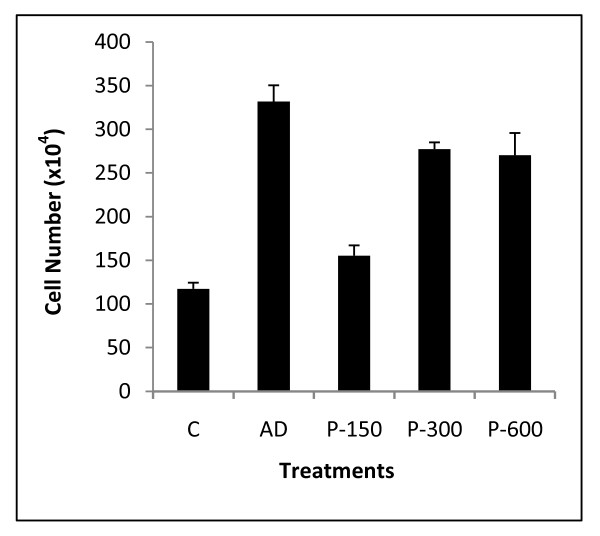
**GP88 induces AC1 cell proliferation *in vitro***. 5 × 10^4 ^cells/plate (35 mm well plates) MCF-7AC1 cells were plated in PRF medium supplemented with 5% Charcoal treated FBS overnight. The next day, medium was removed and serum free medium was added without or with increasing concentration of GP88: 150 ng/ml (P-150), 300 ng/ml (P-300) and 600 ng/ml (P-600) or 25 nM androstenedione (AD). After 6 days, cells were trypsinized and cell number was determined by counting cells with a hemocytometer. Experiment was performed three times in triplicate.

**Figure 2:**
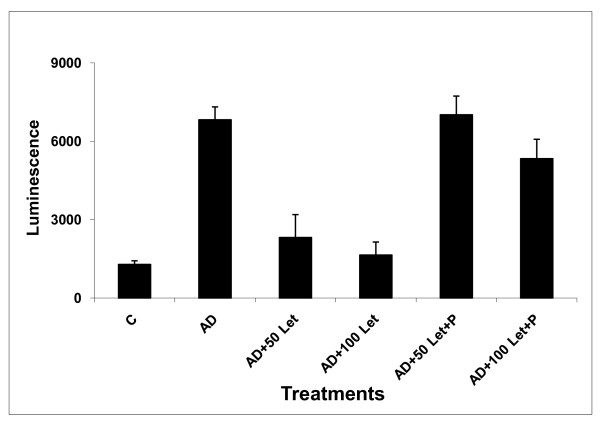
**GP88 confers AC1 cells Letrozole resistance *in vitro***. 2000 cells/well (96 well plates) MCF-7AC1 cells were plated in PRF medium supplemented with 5% ChX FBS overnight The next day, medium was removed and serum free medium was added with and without 300 ng/ml GP88 (GP88 (P)), 25 nM androstenedione (AD) and letrozole (L) 50 nM and 100 nM, 200 nM. Cell proliferation was measured after 6 days using Cell Titer-Glo assay. Experiment was performed in triplicates with p < 0.01.

We examined the effect of human GP88 on proliferation and letrozole responsiveness of MCF-7 AC1 human breast cancer cells overexpressing aromatase. As shown in figure 1, GP88 added at concentrations from 150 ng/ml to 600 ng/ml stimulated the proliferation of MCF-7AC1 cells, maintained in steroid-depleted conditions in a dose-dependent fashion. Stimulation of proliferation by GP88 reached a plateau at 300 ng/ml with a 2-fold increase over control, (p < 0.01) compared to 2.6-fold observed with 25 nM androstenedione (AD) (p < 0.01), the substrate that is converted into estrogen by aromatase enzymatic activity (figure 1). Letrozole treatment of AC1 cells in the presence of AD resulted in a dose-dependent inhibition of growth (figure 2). Addition of 50 nM and 100 nM of letrozole inhibited proliferation by 66% and 76% respectively. Addition of GP88 blocked the inhibitory effect of letrozole (figure 2). In the presence of GP88, 50 nM letrozole had no inhibitory effect on AC1 cells, whereas the inhibitory effect of 100 nM letrozole was reduced from 76% to 22% (p < 0.01).

### GP88 stimulate anchorage-independent growth in aromatase overexpressing AC1 cells

**Figure 3:**
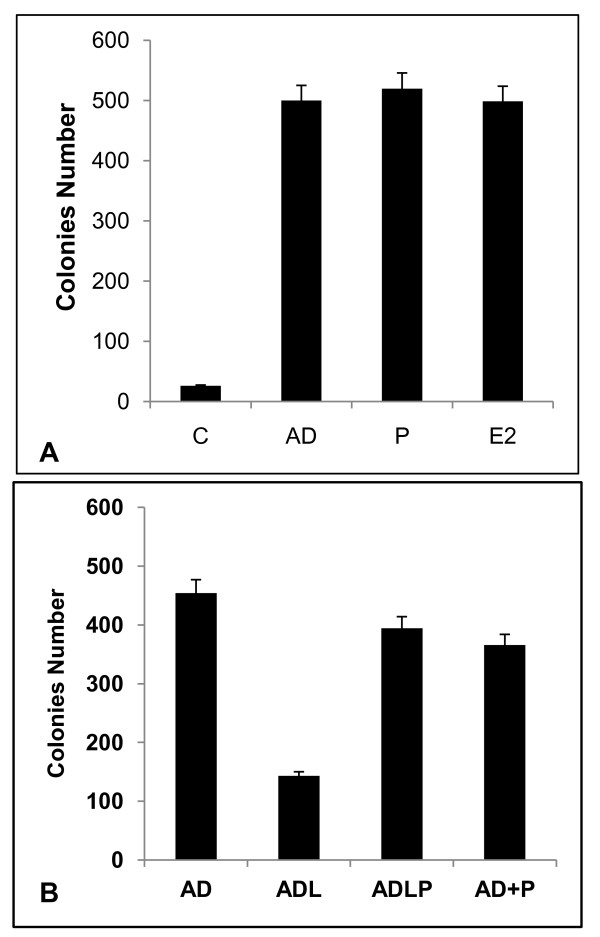
**GP88 induces anchorage independent colony formation and confers letrozole resistance comparable to E2**. A: AC1 cells (1 × 10^4 ^cells/well in 6-well plate) were plated in soft agar plates as described in the Method section in (A) PRF-ChX with and without 300 ng/ml GP88 (P), 25 nM androstenedione (AD) and 200 nM letrozole (L) for 2 weeks. (B) in DMEM/F12 supplemented with 5%FBS, E2 = 10^-8 ^M, 25 nM AD, +/- 300 ng/ml GP88 cultured for 3weeks. Fresh medium was added every 3 to 4 days. At the end of the experiment, colonies were fixed with crystal violet and counted, p < 0.01

Anchorage-independent growth is a hallmark of cancer cell transformation. The ability of exogenously added GP88 to stimulate anchorage-independent growth and confer MCF-7AC1 cells resistance to letrozole was investigated. As described in the method section, cells plated at 1 × 10^4 ^in soft agar in steroid-depleted medium were treated with AD, GP88 or E2 in the absence of letrozole. As shown in figure 3A, addition of GP88 resulted in a 20-fold increase in the number of colonies forming in soft agar (p < 0.01), similarly to what was observed for androstenedione (AD) and for Estradiol, known growth stimulators of aromatase overexpressing breast cancer cells. As shown in figure 3B, addition of letrozole (200 nM) to cells cultivated in the presence of AD resulted in a 53% inhibition of colony growth in soft agar. In addition to stimulating colony growth, GP88 (300 ng/ml) reversed the inhibitory effect of letrozole similarly to E2 taken as control (figure 3A).

### Anchorage-Independent Growth of Stable GP88 transfectants

**Figure 4:**
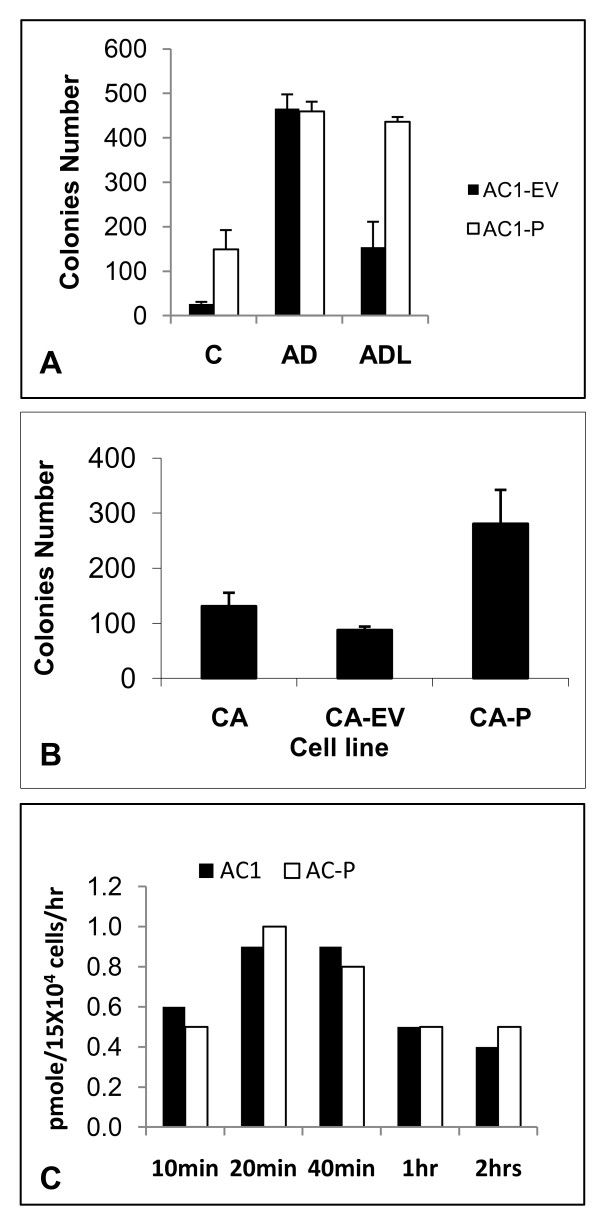
**Overexpression of GP88 in AC1 cells and MCF-7CA cells confer letrozole resistance**. A: GP88 overexpressing AC1 cells (AC1-P) cells were developed by stable transfection of GP88 cDNA expression vector as described in the method section. As control, AC1-EV cells were developed by transfecting empty vector (AC1-EV). For measuring anchorage-independent growth of stably transfected AC1-P cells, AC1-P cells were plated at a density of 5 × 10^3 ^cells/well in a 12 wells plate and cultivated for 2 weeks with/without 200 nM letrozole, 25 nM AD, 300 ng/ml GP88 for the AC1 and CA1-EV in 5% ChX-FBS supplemented medium containing soft agar. Medium was changed every 3-4 days. B: Stimulation of anchorage independent growth of MCF-7-CA cells overexpressing GP88. The aromatase-overexpressing cells MCF-7CA were transfected with expression vector containing GP88 cDNA (CA-P) or with empty vector (CA-EV). Anchorage independent growth of stable transfectants was examined as described above. C: Comparison of aromatase enzymatic activity in AC1 and AC1-P cells. AC1 and AC1-P cells were plated in 6-well plates at a density of 1.5 × 10^5 ^cells/well. Measurement of aromatase activity assay was performed following the protocol described in the method section.

Our data so far show that exogenously-added GP88 induces cell proliferation and anchorage- independent growth in the presence or absence of letrozole. Next, we examined the ability of AC1 cells stably transfected with cDNA coding for human GP88 (AC1-P) to grow in anchorage-independent conditions with or without increasing doses of letrozole. Similarly to the results obtained with exogenously added GP88, AC1-P cells plated in soft agar yielded an increased number of colonies in PRF-ChX medium when compared to AC1-EV cells that did not overexpress GP88. Moreover, addition of letrozole did not decrease colony formation in AC1-P cells in contrast to its inhibitory effect observed with transfected empty vector AC1 cells used as controls (figure 4A). Interestingly, there were no differences in response to AD between the AC1-EV and AC1-P cell lines indicating that even though the cells overexpressing GP88 could proliferate in steroid-free medium, they remained responsive to AD (figure 4A). Similarly to what was observed with AC1 cells, overexpression of GP88 in another aromatase overexpressing cell lines MCF-7CA cells also resulted in a stimulation of growth in soft agar in steroid-depleted medium in the absence of AD (figure 4B).

### GP88 does not affect aromatase enzymatic activity

The ability of GP88 overexpressing AC1 cells to respond to androstenedione suggests that the activity of aromatase was not affected by GP88. However, to verify that GP88 overexpression had no effect on aromatase, aromatase enzymatic activity was measured in the AC1, and AC1-P cells as described in the method section. There were no differences in aromatase activity between the cells AC1 and AC1-P cells (figure 4C). This indicates that expression of GP88 has no effect on aromatase enzyme activity in AC1-P cells.

### GP88 blocks bcl-2 down regulation induced by letrozole

**Figure 5:**
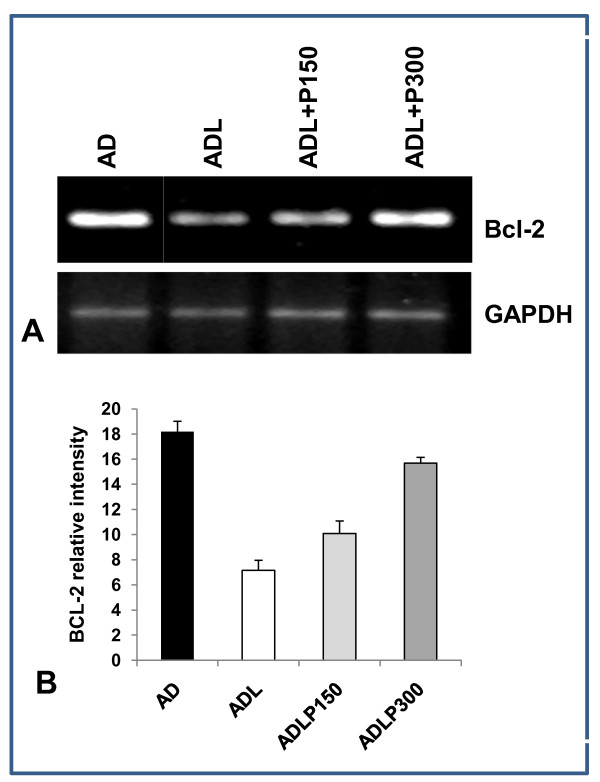
**Bcl-2 upregulation by GP88 in AC1 cells**. A: The ability of letrozole to down regulate bcl-2 expression was examined for AC1. AC1 cells were plated at 1.5 × 10^5 ^cells/well in 6-well plate and incubated in PRF medium with 5% ChX-FBS. The next day, the medium was removed and fresh PRF-5%ChX-FBS medium was added with the indicated treatments 25 nM androstenedione (AD) or AD with 200 nM letrozole (ADL) alone or in the presence of GP88 (150 ng/ml and 300 ng/ml). After 48 hours, total RNA was extracted with Trizol™. Bcl-2 mRNA expression was determined by RT-PCR and examined by agarose gel electrophoresis and staining by ethidium bromide as described in the method section. GAPDH mRNA expression was examined as internal control for equal loading. B: Bcl-2 and GAPDH band intensities of triplicate experiments were determined by densitometry scanning using a UVP gel densitometer. Relative average intensity of bcl-2 bands from the triplicate experiments in each experimental group were then normalized using GAPDH as an internal standard and expressed as bars ± SD, p < 0.05.

Bcl-2 is a key apoptosis regulator that is down regulated in many cell types in response to treatment leading to activation of apoptosis [37]. Bcl-2 expression was examined in AC1 and AC1-P cells treated with androstenedione with and without letrozole. As expected, AD stimulated bcl-2 expression. Addition of letrozole induced the down-regulation of bcl-2 protein expression in AC1 cells (figure 5A). Addition of GP88 prevented bcl-2 down regulation in a dose-dependent manner when compared to cells treated with AD and letrozole (figure 5A). Figure 5B, provides a quantification of the results from triplicate experiments and shows that letrozole inhibited bcl-2 mRNA expression by 61%, whereas inhibition of bcl-2 expression by letrozole was only 24% in the presence of 300 ng/ml GP88 (p < 0.05).

### Letrozole resistant breast cancer cells overexpress GP88

We then examined GP88 expression in cells made naturally resistant to letrozole. Two cell lines were used. The first one termed Long Term Letrozole Treated tumor cells (LTLT) derives from a tumor developed by MCF-7CA cells injected into nude mice following long term exposure to letrozole [23]. The LTLT cells can proliferate in the continuous presence of 1 μM letrozole [38]. The second cell line was AC1-LetR cells derived from AC1 cells that were rendered resistant to Letrozole by cultivating AC1 cells for prolonged periods of time in the continuous presence of 5 nM letrozole.

**Figure 6:**
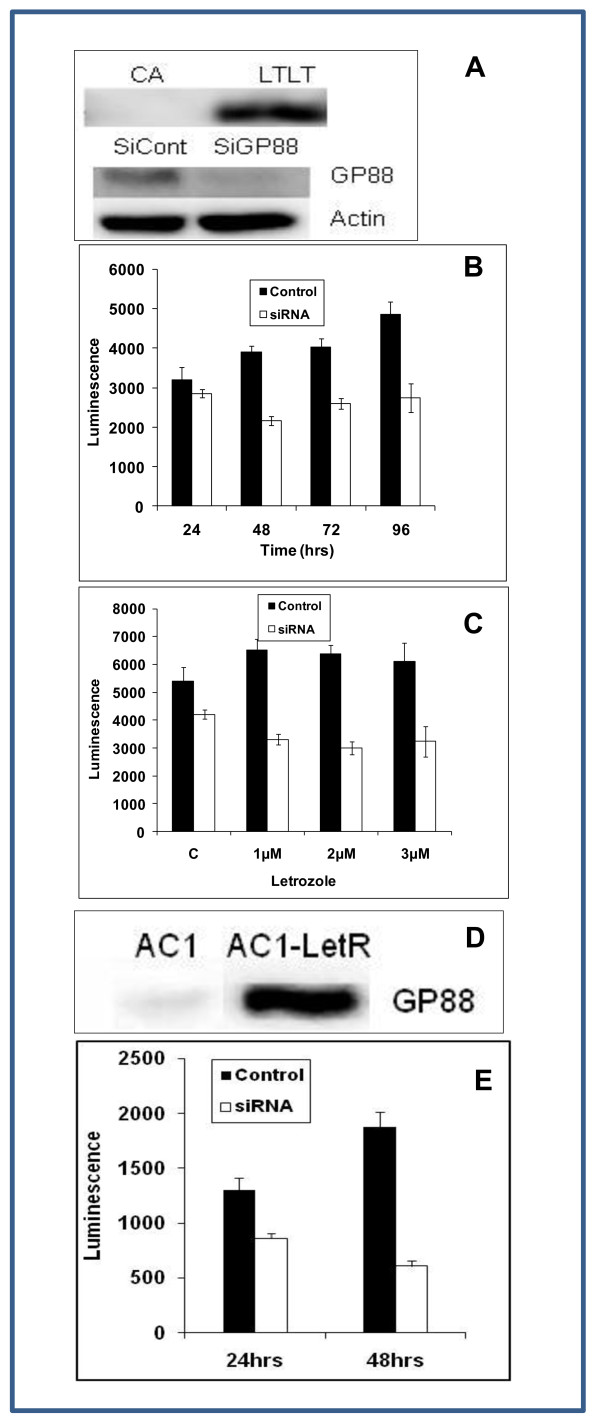
**Effect of silencing GP88 expression on cell proliferation and letrozole responsiveness**. A: Top panel: Comparison of GP88 expression in MCF-7CA cells (letrozole sensitive) and LTLT cells (letrozole resistant cells). Bottom panel: LTLT-CA cells were treated with either siRNA control or with GP88 siRNA for 48 hours before harvesting the cells to determine GP88 expression. GP88 level was determined by IP-Western analysis and normalized to cell number as described previously (Lu and Serrero, 2001). (B) LTLT-CA cells were transfected with GP88 siRNA or control siRNA according to manufacturer's instructions one day before being plated at 2 × 10^3 ^cells/well in 96 well plates in PRF-5%ChX medium and incubated for the time indicated. Cell proliferation was then measured by using the cellTiter-Glo^R ^assay using a luminometer (Molecular Devices). (C) LTLT-CA cells were transfected with either GP88 siRNA or Control siRNA as described above. The next day, cells were plated at 2 × 10^3 ^cells/well in 96-well plate and incubated in PRF-ChX medium for 24 hrs more. After 24 hrs, the medium was removed and cells were washed with PRF medium twice. Cells were then incubated in serum free fresh medium with the indicated concentration of letrozole for 3 days. After 3 days cells were counted using cellTiter-Glo assay, p < 0.05. D) Increase of GP88 expression in AC1-LetR cells derived from AC1 cells rendered letrozole resistant by long term culture in the presence of 5 nM letrozole. E) Effect of silencing GP88 on proliferation of AC1-LetR cells. AC1-LetR cells were transfected with either control siRNA or GP88 siRNA as described in the method section. Proliferation was measured after 24 and 48 hours exposure by cellTiter-Glo^R ^assay, p < 0.05.

We show that LTLT cells express higher level of GP88 when compared to the letrozole sensitive parent cell line MCF-7 CA (figure 6A). GP88 level secreted by the cells was also measured by GP88 EIA developed in our laboratory. LTLT cells had a 13.7-fold increase in the level of GP88 secreted when compared to the letrozole sensitive parent cell line MCF-7CA. The level was 0.8 ± 0.07 ng/10^6 ^cells in MCF-7CA and 11 ± 2 ng/10^6 ^cells in LTLT cells, respectively.

### Silencing GP88 by siRNA reduces cell viability and restores sensitivity to letrozole

The above result shows that GP88 overexpression in LTLT cells is associated with letrozole resistance. We investigated the relation between high GP88 expression and proliferation and letrozole responsiveness of LTLT cells. To answer this question LTLT cells were transfected with SiRNA for GP88 and control SiRNA (1 μM). The level of GP88 in transfected cells was tested both by EIA and western blot analysis. Data from figure 6A (bottom panel) showed that GP88 expression was significantly down regulated within 24 hrs after GP88 SiRNA transfection. LTLT cell proliferation was inhibited by transfection with GP88 SiRNA when compared to cells transfected with non specific control SiRNA (figure 6B). Moreover, GP88 SiRNA treatment of LTLT cells resulted in a 50% growth inhibition upon addition of various doses of 1-3 μM letrozole in contrast to the cells transfected with control SiRNA that showed no response to letrozole (figure 6C). These data show that inhibition of GP88 expression significantly restored letrozole sensitivity of LTLT cells.

We then examined whether GP88 overexpression was also found in other breast cancer cell lines that are resistant to letrozole. As mentioned above, we developed a letrozole resistant cell line named AC1-LetR by continuous cultivation of AC1 cells in the presence of 5 nM letrozole. We also show here that the AC1-LetR cells overexpress GP88 (figure 6D). This overexpression was also quantified by GP88 EIA. The results indicate that GP88 expression was 30-fold higher in AC1-LetR cells (23 ± 2 ng/10^6 ^cells) than in the letrozole sensitive AC1 cells (0.7 ± 0.2 ng/10^6 ^cells). Silencing of GP88 by SiRNA in AC1Let-R cells prevented the cell from proliferating when compared to AC1-LetR cells transfected with control siRNA (figure 6E), similarly to what was observed with the LTLT cells (figure 6A-C).

## Discussion

Development of drug resistance is a recurring problem in treating ER^+ ^breast cancer patients even with the recently introduced aromatase inhibitors [8-9; 36-40]. Overexpression of several growth factors and growth factor receptors overexpression and cross talk with ER have been described as some of the major changes as associated or causing such resistance [17,22,39]. We show here that an alternate growth/survival factor GP88 (progranulin), previously identified as playing a role in breast cancer tumorigenesis [28,29], is involved in conferring letrozole resistance using two aromatase overexpressing breast cancer cells as study models. The present study demonstrates that exogenously added GP88 induces AC1 and MCF-7CA growth in steroid-depleted medium in both anchorage-dependent and independent conditions. In addition, Letrozole induced down regulation of bcl-2 expression whereas GP88 prevented such down regulation. We have also measured viability of the cells treated with androstenedione only (AD), with AD and letrozole (ADL) and with AD, letrozole and GP88 (ADLP). Letrozole reduced AC1 cell viability by 50% whereas addition of GP88 to letrozole treated cells restored viability to the level found on AD treated cells (data not shown).

Interestingly, GP88 cells treated with GP88 or stably overexpressing GP88 formed larger and more numerous colonies in soft agar in steroid-depleted medium even in the absence of androstenedione, the substrate that is converted to estrogen by aromatase. In fact, GP88 had similar growth stimulatory activity as androstenedione or estradiol. Moreover, it was demonstrated that two letrozole resistant cell lines, AC1-LetR and LTLT-CA cells, overexpressed GP88 by 30-fold when compared to letrozole sensitive cells, thereby strongly suggesting a direct relationship between GP88 overexpression and development of resistance to letrozole.

Blocking GP88 expression by SiRNA transfection results in inhibition of cell proliferation in cell lines, AC1-LetR and LTLT-CA cells. We also demonstrate that SiRNA treatment partially restores LTLT-CA cells sensitivity to letrozole. These data provide additional support to our previous findings that GP88 could substitute for estradiol to stimulate proliferation, in steroid-depleted medium and confer tamoxifen resistance to MCF-7 cells both in vitro and in vivo [32]. Our previous pathological study with paraffin-embedded breast cancer biopsies has shown that GP88 is expressed in ER^+ ^invasive ductal carcinoma (IDC) in association with poor prognosis whereas GP88 expression was negative in benign lesions and normal mammary epithelial tissues [31] supporting the fact that GP88 could play a role in clinical outcomes of breast cancer patients. Moreover, we showed by a retrospective clinical study that high GP88 expression measured by IHC in tumor biopsies of patients with ER^+ ^IDC was associated with a significant decrease in disease-free and overall survivals (Serrero et al, submitted to publication). In addition, a recent published study has shown that GP88 also plays a role in triple negative breast cancer (ER, PR and Her2 negative) with high GP88 expression associated with poor survival outcome [41].

Overexpression of growth factors and, growth factor receptors will make aromatase overexpressing cells able to by-pass the need of estradiol for proliferation. Several mechanisms have been hypothesized, in particular cross-talking with ERα and/or constitutive activation of MAPK leading to the down regulation of ERα, making the tumors insensitive to estradiol and thus to AIs therapies [23,40]. Such possibilities have been described for the letrozole resistant cell line LTLT cells that have been isolated from tumor treated with letrozole for a prolonged period of time [23]. These cells were found to have lower level of ERα and higher Her-2 level than their letrozole sensitive counterparts MCF7-CA cells. The cells appear to survive estrogen deprivation by the activation of Her-2/MAPK pathway [42]. In fact, for these letrozole resistant cells, the combination of letrozole and trastuzumab resulted in superior outcome in inhibiting tumor growth in vivo over letrozole or trastuzumab alone leading to inhibition of tumor growth, in contrast to MCF-7CA cells where letrozole alone was as effective as trastuzumab in combination with letrozole. This indicates that overexpression of Her-2 is a major contributor of letrozole resistance of LTLT cells. We show here that the autocrine growth factor GP88 confers letrozole resistance to MCF-CA cells and that in LTLT cells that overexpress GP88, inhibition of GP88 expression by SiRNA partially restores letrozole responsiveness. In addition, GP88 also conferred letrozole resistance to AC1 cell line, another aromatase overexpressing cell line. Concerning the possible mechanisms by which GP88 confers letrozole resistance, we show here that AC1 cells overexpressing GP88 can still be stimulated by androstenedione (figure 4A) in steroid-depleted medium and by estradiol ( data not shown) indicating that the letrozole resistance caused by GP88 is not due to the cells becoming estrogen independent or not expressing estrogen receptors. Moreover, as shown in figure 4C, aromatase enzymatic activity was not affected by GP88 overexpression thereby suggesting that GP88 expression did not result in a defect in aromatase expression or function. The fact that GP88 has been shown to be a potent proliferation and anti-apoptotic factor for breast cancer cells [26,33,34] would suggest that GP88 confers resistance to the killing effect of letrozole by providing growth and survival advantage to the cells rather than by blocking estrogen response or by inhibiting aromatase activity.

It is also interesting to note that we have shown previously that GP88 activates Her-2 phosphorylation in breast cancer cells through the ERK1/2 and Akt pathways [43]. This would suggest the association of GP88 and Her-2 in conferring resistance to letrozole. However, our pathological studies with breast cancer tumor biopsies have indicated that GP88 and Her-2 were independent biomarkers [31], suggesting that not all of GP88 effects on letrozole resistance are necessarily mediated via GP88's ability to cross-talk with Her-2.

## Conclusions

Understanding the role and mechanisms of GP88 in the development of resistance to anti-hormonal treatments could contribute to improving the treatment of breast cancer. Based on our pathological studies, it would be interesting to examine if overexpression of GP88 in tumor tissue s is associated with poor response to AI. Moreover, combination of drugs that include the inhibition of growth factors and anti-hormones could provide new avenues to treat breast cancer patients and overcome resistance. Our findings provide a novel paradigm for the acquisition of aromatase inhibitor resistant breast cancer and could suggest new therapeutic approaches.

## List of Abbreviations

AD: androstenedione; AI: aromatase inhibitors; E2: 17-β estradiol; ER: estrogen receptor; FBS: fetal bovine serum; ChX-FBS: charcoal extracted fetal bovine serum; Let: letrozole; PRF medium: phenol red free medium; PRF-ChX: phenol red free DME-F12 medium supplemented with 5% Charcoal extracted fetal bovine serum; SDS: sodium dodecyl sulfate; PAGE: polyacrylamide gel electrophoresis

## Competing interests

Drs. Ginette Serrero, Changsheng Tian and Ms. Binbin Yue have financial competing interests as employees and stock holders of A&G Pharmaceutical Inc. Drs. Tesfom Abrhale, Angela Brodie, Luciana Macedo, and Gauri Sabnis do not have competing interests.

## Authors' contributions

TA carried out the GP88 studies and drafted the manuscript. AB, and LM developed the cell lines used in these studies and helped in data analysis and thoroughly reviewed and revised the manuscript. GaSa carried out the aromatase studies and helped in data input and data analysis. CT designed and develop conditions for the SiRNA experiments and the RT-PCR experiments. BY did all the EIA GP88 measurements for all cell lines studied. GS conceived the study, drafted the manuscript, directly guided TA in his studies and provided the input on experimental design and data interpretation.

All authors have given final approval of the submitted manuscript.

## Pre-publication history

The pre-publication history for this paper can be accessed here:

http://www.biomedcentral.com/1471-2407/11/231/prepub
